# A Natural Botanical Product, Resveratrol, Effectively Suppresses Vesicular Stomatitis Virus Infection In Vitro

**DOI:** 10.3390/plants10061231

**Published:** 2021-06-17

**Authors:** Shih-Chao Lin, Xiang Zhang, Caitlin W. Lehman, Han-Chi Pan, Ya Wen, Shiow-Yi Chen

**Affiliations:** 1 Bachelor Degree Program in Marine Biotechnology, College of Life Sciences, National Taiwan Ocean University, Keelung 20224, Taiwan; sclin@mail.ntou.edu.tw; 2 Department of Molecular Medicine and Surgery, Karolinska Institutet, SE-171 77 Stockholm, Sweden; xiang.zhang@ki.se; 3 Department of Biomedical Sciences and Pathobiology, Virginia-Maryland College of Veterinary Medicine, Virginia Polytechnic Institute and State University, Blacksburg, VA 24061, USA; Woodsonc@vt.edu; 4 National Center Animal Laboratory, National Applied Research Laboratories, Taipei 11599, Taiwan; hcpan@narlabs.org.tw; 5 Department of Physiology and Pharmacology, Karolinska Institutet, SE-171 77 Stockholm, Sweden; ya.wen@ki.se; 6 Department of Bioscience and Biotechnology, National Taiwan Ocean University, Keelung City 20224, Taiwan

**Keywords:** resveratrol, vesicular stomatitis virus, antiviral activity, veterinary science, husbandry farming

## Abstract

Numerous natural phytochemicals such as resveratrol are acknowledged as potent botanical agents in regulating immune responses. However, it is less understood whether such immunomodulatory phytochemicals are appropriate for use as direct treatments in veterinary viral diseases. In the present study, we investigated the efficacy of resveratrol in suppressing vesicular stomatitis virus (VSV) infection. Outbreaks of VSV can cause massive economic loss in poultry and livestock husbandry farming, and VSV treatment is in need of therapeutic development. We utilized a recombinant VSV that expresses green fluorescent protein (GFP) to measure viral replication in cells treated with resveratrol. Our findings revealed that resveratrol treatment affords a protective effect, shown by increased viability and reduced viral replication, as indicated by a reduction in fluorescent signals. Additionally, we found that resveratrol inhibition of VSV infection occurs via suppression of the caspase cascade. Structural analysis also indicated that resveratrol potentially interacts with the active sites of caspase-3 and -7, facilitating antiviral activity. The potential effect of resveratrol on reducing VSV infection in vitro suggests that resveratrol should be further investigated as a potential veterinary therapeutic or prophylactic agent.

## 1. Introduction

Vesicular stomatitis virus (VSV) is a bullet-shaped, enveloped, negative-sense, single-stranded RNA virus, and is a member of the *Rhobdaviridae* family [1,2]. VSV is also an arbovirus, transmitted primarily by sand flies or black flies. The sand fly species *Lutzomyia shannoni* can persistently harbor and support VSV replication without exhibiting lethal diseases or cytolytic effects [3]. Two principal serotypes of VSV, New Jersey and Indiana, are known to be infectious to chickens, bovines, equines, and swine, causing systemic symptoms such as anorexia, lethargy, and pyrexia akin to foot and mouth disease in cloven-hooved ruminants [4]. In addition to arthropod-borne transmission, VSV also swiftly spreads within herds through direct contact with infected animals or fomites [5]. Occasionally, VSV infection in farm animals leads to spillover events in the human population, particularly in farmworkers who are in close contact with infected animals. In humans, symptoms are primarily flu-like, including fever, muscle ache, fatigue, and oral blisters. On rare occasions, children can develop encephalitis, as VSV is capable of infecting dendrites of hippocampal neurons [6,7]. As a result, it is critical to contain and eliminate VSV outbreaks within the animal population in order to minimize economic impacts, as well as prevent the spread of this zoonotic infectious disease within the human population.

VSV is a highly cytopathic virus that causes robust infection and has been known to induce strong immune responses on infection [8]; as such, modified forms of VSV are frequently utilized as a carrier when developing vaccines against other lethal pathogens such as the Ebola virus or SARS-CoV-2 [9,10]. VSV infection of Hela cells induces >90% of a cytopathic effect (CPE) within 12 h post-infection [11]. VSV also elicits profound humoral and cellular immunity, particularly the CD8 T cell-mediated response [12]. However, this overwhelming CD8 T cell response appears to play a role in VSV-induced pathogenesis. Susceptible BALB/c mice with MHC-I-deficiency seemed to be more resistant to lethal infection of VSV, suggesting the nonspecific and uncontrolled immunity could contribute to the immunopathogenesis [13]. Here, we sought to utilize an immunomodulatory phytochemical to investigate the potential of inhibition of VSV in vitro.

Resveratrol, a polyphenolic stilbenoid, exists in various esculents, such as cocoa, peanuts, soy, and grapes, but can be most abundantly found in red wine made from grape skins and vaccinium berries, such as bilberries, blueberries, and cranberries [14]. *Trans*-resveratrol glucoside, a metabolite of resveratrol found in certain herbal plants such as Japanese knotweed (*Fallopia japonica*), can serve as an ingestible form of resveratrol [15]. Resveratrol’s most famous bioactivity is its antiaging effects via mitochondrial activation in animals [16]. However, its antiaging activity is highly dependent on the model organism and may only be marginal [17,18]. As such, recent studies have focused more on its inhibitory activity against inflammation, oxidation, cancers, and microbial infections [19,20]. Specifically, resveratrol has been shown to regulate inflammatory responses via activation of Sirt1 and subsequent downregulation of NF-κB expression [21,22]. Due to its wide range of bioactivity, resveratrol has been recognized as one of the most famous flavonoids. Given that resveratrol has been shown to facilitate protective effects of host cells against various viral infections [23,24,25], we therefore aimed to evaluate resveratrol for anti-VSV activity and investigated the possible mechanisms underlying its antiviral activities.

## 2. Materials and Methods

### 2.1. Chemicals

Resveratrol (trans-3,5,4′-trihydroxystilbene; cat.# P5010) and phloretin (cat.# P7912) powder were purchased from Sigma-Aldrich (St. Louis, MO, USA) with a purity of ≥99%. Resveratrol or phloretin was initially dissolved in dimethyl sulfoxide (DMSO) and filtered with a 0.22 μM syringe filter to remove potential microbial contamination and then cryopreserved at –80 °C until further usage. Prior to the treatment of cells, resveratrol or phloretin was diluted in DMEM supplemented with 2% FBS and serially diluted to working concentrations.

### 2.2. Cell Lines and Cell Culture

The Vero cell line (African green monkey kidney cells) was a generous gift from Dr. Ya-Fang Wang at National Health Research Institute (Taiwan), and the MRC-5 cell line (CCL-17, human lung fibroblast cells) was purchased from ATCC (Manassas, VA, USA). Both cells were cultured in Gibco^TM^ high glucose DMEM (ThermoFisher Scientific, PA, USA) supplemented with 10% FBS and were maintained in a 37 °C incubator with 5% CO_2_.

### 2.3. Virus Infection

Vero or MRC-5 cells were seeded in 96-well plates at a concentration of 4 × 10^4^ cells per well overnight. Prior to infection, cells were pretreated with indicated concentrations of resveratrol or phloretin for 1 h, followed by infection of VSV at a multiplicity of infection (MOI) of 0.1 at 37 °C. Following washing off the viral inoculum, complete culture media containing fresh resveratrol was added back to cells and incubated for an additional 24 h post-infection (hpi).

### 2.4. Cell Viability Assay

Cells were seeded in 96-well plates and infected with VSV at an MOI of 0.1 for 24 h with or without resveratrol or phloretin. At 24 hpi, cellular supernatant was removed, and luciferase activity was measured using a CellTiter-Glo Luminescent Cell Viability assay (Promega, Madison, WI, USA) per the manufacturer’s instructions. Luminescence was read on a luminescence reader to obtain the relative luminescence unit (RLU), which is highly correlated to the concentration of cellular ATP [26]. The percentage of cell viability was calculated relative to the uninfected or untreated control cells.

### 2.5. Fluorescence Analysis

Vero cells were infected with GFP-expressing VSV at an MOI of 0.1 for 24 h following seeding on 12-well plates the day before. Prior to visualization of the fluorescent signals, cells were fixed with 4% paraformaldehyde for 20 min at room temperature and then washed twice with PBS. Cells were permeabilized with PBS solution containing 0.1% Triton X-100 for 15 min and stained with 10 μg/mL of propidium iodide (PI) in PBS for 10 min. After washing the PI staining, the fluorescent signals were observed via EVOS Cell Imaging System (ThermoFisher Scientific, PA, USA), and the fluorescent images were merged using ImageJ software to better reveal the locations of replicated VSV [27]. For detecting dead cells in PI uptake assays, the same protocol was applied as previously described without permeabilization.

### 2.6. Plaque Assay

The protocol of plaque assay was adapted from a previous study with minor modifications [28]. Briefly, the supernatant of VSV-infected cells was collected and serially diluted in a 10-fold manner prior to the infection of Vero cells. After incubation, inoculums were removed and cells were washed once with PBS, followed by the addition of an overlay medium made with 2% agarose and 2X EMEM supplemented with 5% FBS, sodium pyruvate, and non-essential amino acids (NEAA). Plates were fixed with 10% formalin after 48 h incubation at 37 °C, 5% CO_2_ incubator, and stained with 0.2% (*w*/*v*) crystal violet to visualize and count plaques, which were used to calculate viral titers.

### 2.7. Docking Prediction

The structure of chain a and chain b were cleaved from caspase-7 (PDB: 3IBF) by PyMOL, and the whole structure of caspase-3 (PDB: 1QX3) was used to perform blind molecular dockings with resveratrol (ZINC6787) by SwissDock [29]. The putative binding regions on caspase-3 and -7 were evaluated by the SwissDock web server, based on the vicinity of all target cavities as a blind docking. The docking results were visualized by PyMOL and UCSF Chimera v1.15 [30] via space-filling models. The molecular interactions and distances of hydrogen bonds were plotted by LigPlot v2.2.4 [31]. The lowest free energy (ΔG) generated from SwissDock was regarded as the strongest binding interaction between caspases and resveratrol.

### 2.8. Caspase-Glo^®^ 3/7 Assay

Cells were infected with VSV (or left uninfected) and treated with resveratrol or solvent control and analyzed for caspase-3 and -7 activities by the Caspase-Glo^®^ 3/7 assay system (Promega^®^), which is also a luminescent-based assay, per the manufacturer’s instructions [32]. Briefly, the caspase-3/7 Z-DEVD-aminoluciferin substrate and cell lysis buffer were mixed in a 1:1 ratio to allow the activation of luciferase, which converts the caspase-3 and -7 activities into luminescent signals for quantification with a luminescence plate reader.

### 2.9. Statistical Analysis

Statistical significance, defined as P values below 0.05, was calculated and compared after performing one-way ANOVA by software GraphPad Prism unless stated otherwise.

## 3. Results

**Figure 1:**
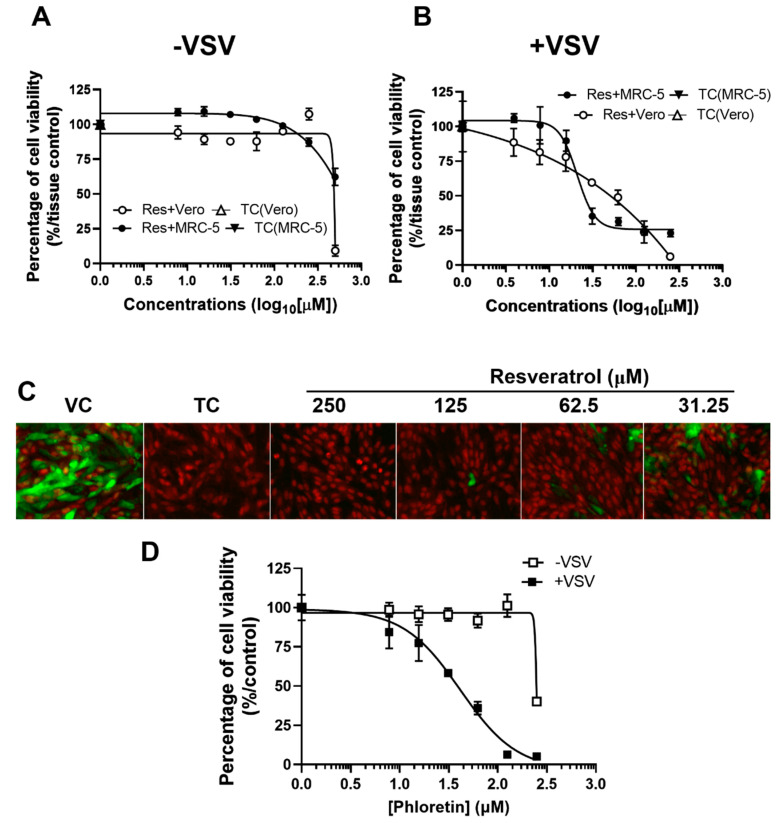
Determination of CC50 and EC50 of resveratrol. Vero and MRC-5 cells were treated at indicated concentrations of resveratrol (**A**) without or (**B**) with VSV infections at an MOI of 0.1. The cell viability was measured by CellTiter-Glo assays at 24 hpi and the (**A**) CC50 and (**B**) EC50 values were calculated and denoted on each panel. (**C**) VSV replication in Vero cells with or without resveratrol was visualized through the GFP signals with the propidium iodide staining for nuclei by EVOS fluorescence microscope. (**D**) CC50 and EC50 of phloretin against VSV was tested on Vero cells at an MOI of 0.1 at 24 hpi. All experiments were performed in triplicate, and data are presented as mean ± SEM: TC, tissue control; VC, viral control.

The cytotoxic effects of resveratrol on Vero and MRC-5 were determined in the absence of VSV infection (Figure 1A) to determine the median cytotoxicity concentration (CC50), as well as in the presence of VSV infection (Figure 1B) to determine the median effective concentration (EC50). Resveratrol exhibited very limited cytotoxicity until the concentration reached above 500 μM for both Vero and MRC-5 cells (Figure 1A). The CC50 of resveratrol was 509 μM on Vero cells and, comparably, 465.5 μM on MRC-5 cells after 24 h of treatment. The CC50 values indicate that treating mammalian cells with resveratrol did not induce significant cytotoxicity.

Following the determination of cytotoxicity, we continued to evaluate effective concentrations of resveratrol against VSV at 24 hpi using CellTiter-Glo assays (Figure 1B). Luminesce values indicate the EC50 for resveratrol in Vero cells was 74.5 μM and 19.3 μM in MRC-5 cells, and rendering therapeutic indices (TI) were calculated to be 6.8 (Vero) and 24.1 (MRC-5), respectively. These data suggest that resveratrol possesses antiviral activity against VSV infection with limited cytotoxicity at lower concentrations, especially on normal human lung cells.

We further confirmed the antiviral activity of resveratrol against VSV with a GFP-expressing recombinant VSV (Figure 1C). Vero cells were fixed with 4% paraformaldehyde and stained with propidium iodide (PI) to visualize the cell nuclei. As shown in Figure 1C, VSV actively replicates in Vero cells, revealing strong GFP signals in the cytosol but not in the nuclei. However, GFP fluorescence intensity was remarkably reduced upon resveratrol treatment in a dose-dependent fashion, even at lower concentrations of resveratrol (31.25 μM), demonstrating that resveratrol is potent and effective in suppressing VSV replication. For comparison, we treated Vero cells with a PKC inhibitor, phloretin, which has been previously shown to reduce VSV replication [33]. Results indicated that CC50 of phloretin on Vero cells was 245 μM, which is slightly more toxic than resveratrol; however, the EC50 of phloretin was 46.7 μM (Figure 1D), suggesting that anti-VSV efficacy of resveratrol is comparable to another antiviral phytochemical.

**Figure 2:**
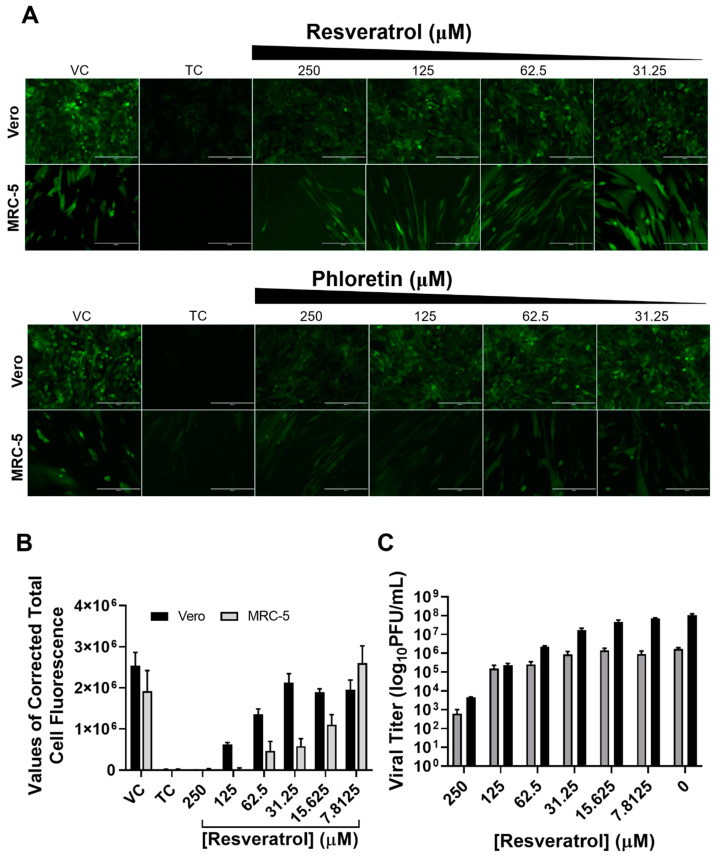
Inhibition of VSV propagation by resveratrol and phloretin. Vero and MRC-5 cells were infected with VSV-GFP recombinant virus: (**A**) Cells were subsequently examined for GFP intensity with various concentrations of indicated compound. (**B**) Quantification of fluorescence intensity as corrected total cell fluorescence (CTCF) from at least three randomly-selected cells by ImageJ software. (**C**) Culture media from infected cells were collected for viral titration via plaque assays. The VSV titers in log10 scales were presented as mean ± SEM from triplicate experiments.

Further, we examined the GFP signal of VSV in Vero and MRC5 cells with decreasing resveratrol treatments after infection at MOI of 0.1 for 24 hpi (Figure 2A,B). Fluorescence microscopy shows that the VSV-GFP efficiently infected Vero and MRC-5 cells, as nearly every cell expressed GFP by 24 h after infection. Despite the swift and vigorous infection, resveratrol could effectively reduce the VSV-GFP infection in a dose-dependent manner in both cell lines, as further demonstrated by quantification of total cell fluorescence by ImageJ software [34] (Figure 2B). Viral titers in the culture supernatant indicate that, even though Vero cells were more permissive to VSV infection than MRC-5, most likely due to the lack of type I interferon [35], resveratrol can inhibit VSV replication from reducing, as shown by the reduction in viral titers by 4- (Vero) and 3- (MRC-5) logs (Figure 2C).

**Figure 3:**
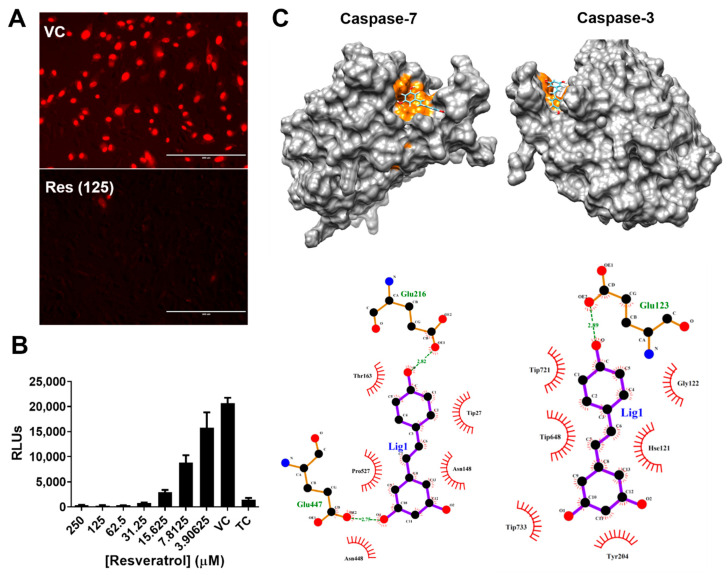
Caspase cascade was affected by resveratrol: (**A**) Cell death-induced VSV infection was observed by PI uptake assay. The intercalating PI indicators inside nuclei were excited and visualized by EVOS fluorescence microscope. (**B**) The caspase-3 and -7 activities of Vero cells were measured following VSV infections (MOI = 1) at 24 hpi by Caspase-Glo 3/7 assay system. All experiments were performed in triplicate, and data were plotted as mean ± SEM: TC, tissue control; VC, viral control; RLU, relative light unit. (**C**) Computational docking toward the docking predictions between resveratrol and caspase-3 and -7 were performed via SwissDock, and the results were visualized by UCSF Chimera software. The active sites of caspase-3 and -7 are highlighted in orange.

Given that VSV infection induces massive cell death through the caspase-3-associated apoptosis pathway [36], it is critical to elucidate whether resveratrol exerts its anti-VSV activity via caspase signaling. To this end, we first utilized the propidium iodide uptake assay to detect apoptotic cells based on the integrity of the plasma membrane [37]. The fluorescent images obtained from the PI uptake assay clearly exhibit the two distinct conditions. Vero cells infected with VSV in the absence of resveratrol treatment revealed significant red fluorescent signals in nearly all cells due to the breach of the plasma membrane, indicating a high level of cell death. Conversely, PI uptake was prohibited in cells with intact plasma membranes with resveratrol treatment, suggesting cell viability was increased (Figure 3A). To further supplement this finding, activities of caspase-3 and -7 following VSV infection were measured. Results indicate that VSV induced strong caspase-3 and -7 activities without resveratrol. However, in the presence of resveratrol, these activities were reduced in a dose-dependent fashion in accordance with the increased concentrations of resveratrol (Figure 3B).

To further confirm and elucidate the details of how resveratrol interacts with caspase-3 and -7, we performed in silico docking to assess the interactions between caspase proteins and resveratrol. The docking predictions revealed that the active site of caspase-7 [38] and caspase-3 [39] form hydrogen bonds from a distance of 2.75–2.89 Å with resveratrol (Figure 3C), suggesting that resveratrol could actively bind to caspase-3 and -7 and, thus, interfere with their physiological functions. Altogether, our data suggest that VSV-induced apoptosis was effectively inhibited by resveratrol by restricting the activities of caspase-3 and -7, which could play a pivotal role in anti-VSV activity.

## 4. Discussion

VSV infection is robust and highly contagious. Despite rare cases in humans, VSV outbreaks can cause significant economic loss and animal death within animal husbandry farming. There are limited antiviral drugs available to treat VSV infection, such as ribavirin and chloroquine [40]. However, such antiviral drugs are quite costly when used extensively on infected animals in the field. Like resveratrol, polyphenols, are more economical and can be used as feed additives in the diet to reduce the risks of various infectious diseases [41]. Our current study suggests resveratrol treatment could be useful in preventing, controlling, or treating VSV outbreaks, and it is an attractive target for therapeutic development.

To the best of our knowledge, our study is the first to utilize resveratrol as an anti-VSV remedy. In addition to VSV, resveratrol has been tested on the pseudorabies virus in piglets and resulted in increased production of antiviral cytokines, such as interferon-α and -γ, interleukin 12, as well as tumor necrosis factor-α after 7 days post-infection [42]. Moreover, in piglets, it has been shown that resveratrol could alleviate diarrhea caused by rotavirus via its immunomodulatory functions [43]. As such, the various applications of resveratrol warrant further investigation and could be beneficial in the fields of animal agriculture and veterinary medicine [44].

Although our study is the first investigation of resveratrol as it relates to anti-VSV properties, a previous study has implied the potential use of berry-derived polyphenols in VSV infection. Instead of a single, pure compound, Ahmed et al. [45] exploited an in vitro model to show the cell protection effects of blueberry green tea polyphenol soy protein complexes against VSV infection. In addition to the direct cell death caused by VSV, the neurovirulence aspect of VSV is another major concern [46]. Resveratrol has been shown to exhibit neuroprotection through antioxidative and anti-inflammatory activities [47], but further studies are required to determine whether resveratrol can alleviate the neurological effects associated with VSV.

The highly apoptotic activity of VSV has rendered it an oncolytic candidate for cancer therapies. As such, VSV is currently being tested in an ongoing clinical trial for liver cancer since 2012 (NCT 0162864). The unfolded protein response (UPR) is triggered by VSV infection, which induces apoptosis via the recruitment of caspase-3 and -7 [36,48]. Importantly, this apoptotic activity was reduced with resveratrol treatment (Figure 3). However, the replication of VSV seemed not to trigger the caspase activation per se, according to Hobbs et al. [36]. Therefore, further investigations are required to identify the lytic mechanisms of VSV and the anti-apoptotic activity provided by treatment with resveratrol.

While we observed strong anti-apoptotic activity after treatment with resveratrol (Figure 3B), some other studies have reported that resveratrol is capable of inducing significant apoptosis in particular cell types, which may be independent of sirtuin-1 activation, including pancreatic, colon cancer cells, U937, HL-60, and MOLT-4 leukemia cells [49,50]. However, the discrepancy in apoptosis could likely be due to the cancerous condition versus normal tissues [51].

In conclusion, our findings suggest that immunomodulatory phytochemicals such as resveratrol could effectively inhibit cell death induced by VSV infection. The antiviral functions of resveratrol in terms of VSV infection is likely due to its ability to reduce apoptotic activity induced by viral infection. Thus, resveratrol is worthy of further investigation and development as an anti-VSV agent.

## Data Availability

The data supporting the findings of this study are available within the article.
